# Lunatic Asylums in the United States

**Published:** 1849-11

**Authors:** 


*Lunatic Asylums in the United States. The Pennsylvania Hospital.*

Sir,—A recent visit to the United States suggests a few remarks on the treatment of the insane in that country, which may not be unacceptable to your readers.

The American hospitals for the insane are, generally speaking, much better adapted for the cure and comfort of the patients than similar institutions in this country, The moral treatmant, also, is of comparative excellence, and upon moral treatment alone can any dependence be placed for restoring the healthy functions of the brain, the efficiency of medicines, in the majority of cases, being very questionable.

The first consideration should be, to place the patient in an asylum the internal and external arrangements of which are as nearly like what would be desirable for the sane, as is compatible with their treatment and security. This, of course, implies that there should be the least possible degree of restraint, in reality, as in appearance. Under these circumstances, when combined with moral treatment, and the promotion of physical energy, the best results may be anticipated.

The Pennsylvania Hospital for the Insane, near Philadelphia, is probably better adapted for carrying out this judicious treatment than any other establishment in that country, or elsewhere.

The form of the building, in which all excessive ornament or extravagance has been avoided, is in the best taste, and calculated to produce a pleasing impression on all who see it. The windows are of the ordinary size, the sashes being of cast iron, and of a very light appearance; they are hung in a frame cased with iron, balance each other, so as to be moved with ease, and as one rises the other falls, but only to the extent of six inches, or not sufficient to allow the escape of a patient. With this form of window all bars and screens are dispensed with, the appearance being precisely that of a wooden sash, and it never produces unpleasant feelings. Where it is necessary to protect the glass, a light wire netting is stretched over a frame, which is hinged, and secured by a spring lock. This frame is sometimes separated a few feet from the window, and the space filled with evergreens and flowering plants.

The patients of each sex are divided into six distinct classes; each class occupies a ward, to which are connected a parlor, dining-room, clothes-room, bath-room, and a water closet, a corridor, with chambers on one side, a dormitory, rooms for two attendants, a dumb waiter, and a funnel for soiled clothes, leading to the basement story. In the wards occupied by the lowest patients, the parlors are dispensed with, and special provision is made for the worst class, who are completely isolated from the main building. The associated dormitories are occupied by about one-fourth of the patients. Every part of the hospital is open to the direct rays of the sun, and has a light and cheerful appearance; all possible advantage is derived from natural and forced ventilation. The apartments are sufficiently warmed by hot water and steam, under low pressure, circulating in iron pipes, and this plan has been adopted as the best result of numerous experiments.

The pleasure grounds contain forty-one acres, and are surrounded by a wall ten feet and a half high, a very small portion of which is seen from the building; indeed, no one thinks or speaks of it as a prison enclosure. When we consider how impatient the insane are of detention, and how much mental excitement is allayed by muscular exertion, the importance of extensive grounds and varied scenery cannot be overrated,—such influences are always exerting a silent good on the worst class of patients. Dr. Kirkbride, the resident physician, is of opinion that high walls round small inclosures, and in full view, are worse than none. It is difficult, indeed, to make these airing-courts look otherwise than prison-enclosures,—although, at the Retreat, near York, and at the Bloomingdale Asylum, near New York, much has been done by garden-culture and planting, to obviate this appearance.

Statistical tables are carefully kept at the asylums at Philadelphia, New York, and at Boston; and by a comparison with those of England, it is found that the average number of cures is considerably greater than in that country. It is an established principle, that insanity is curable in proportion to the early period at which it is placed under treatment; and from this circumstance one might conclude that the result would be more favorable in England, where the accommodation is so much more extensive, and the treatment so much more prompt. The inference, however, is, that the system adopted in America is more judicious. With few exceptions, they regard all chronic cases as susceptible of cure, or much improvement; and that large class, usually called incurable, are treated with a perseverance which is often rewarded with a successful issue.

Dr. Kirkbride believes, that of all who are attacked with insanity, and subjected, during its early stages, to judicious treatment, faithfully persevered in, at least eighty per cent. will probably recover. It is impossible to say who are incurable: I have seen one person restored to health, who for many years was kept chained in an out-house, laboring under the worst form of insanity short of actual dementia. This individual became the chief cook of the establishment in which she had been so long confined as a patient. Many cases, believed to be incurable, are placed in confinement for safety only; and thus condemned to the exclusive society of incurables, whereby any chance of returning reason is frustrated. Every intelligent person knows the effect of association upon his own mind: he feels in himself, and sees in others, that moral and refined society have an elevating influence; while the companionship of the profligate, ignorant, and deluded bring down the standard of the mind, and destroy its natural balance. Hence moral means which are to aid in the restoration of curables are often used in keeping up the mental powers of those who may be incurable.

The American tables show, as usual, that a large proportion (nearly one-third) of the patients are admitted between twenty and thirty years of age; this is the period of life during which the passions are most excitable, and the mind most acutely sensible to avarice and ambition; for the like reason, however, these cases are more easily influenced by moral treatment.

The chief excellence of this treatment in the asylums of the United States, consists in carefully classifying the patients, and in arranging the inmates of the separate wards, so as to exclude those cases, the subjects of which may in any way interfere with the progress of the majority; and those who are practically conversant with the treatment of the insane, will admit that this class separation is as imperative for insuring the mental restoration of the insane, as individual seclusion is necessary for improving the moral condition of the criminal.

There is also an individual in the wards of the Pennsylvania Hospital, whose duties are of the highest importance, and whose office, as far as my knowledge extends, is peculiar to that institution. This person is called a companion, or teacher, and is indeed the active and untiring agent of the physician. The companion is entirely relieved from the domestic duties of the keepers, and being intelligent and courteous, is the means of effecting a great amount of good, by teaching the patients what will help to rid them of their delusions, promote their happiness, and hasten their recovery. In our asylums at home, too little attention is paid to the selection of keepers; commonly speaking, any person possessing a kind disposition and physical power is thought to be qualified to attend day and night on the insane; forgetting how essential, also, are tact, and that facility of conversation which is often so soothing to the excited imagination. Great improvements have, however, been made in all that relates to the management of lunatics, much of which is to be attributed to the active control of the legislature; and we may trust the day is not far off when such prison-like structures as St. Luke's and its dungeons will cease to be seen in the land.

I am, Sir, your obedient servant,

*London Lancet.* J. THORNTON, M. R. G. S.

